# Comprehensive assessment of salt tolerance in nine spring maize hybrids across the entire growth period

**DOI:** 10.3389/fpls.2026.1871695

**Published:** 2026-07-20

**Authors:** Yipu Li, Zhenghan Xu, Yanan Lin, Shiyao Wang, Beizhou Wang, Jian Liu, Haizhu Bao, Julin Gao, Xiaofang Yu

**Affiliations:** 1 Agricultural College, Region Research Center for Conservation and Utilization of Crop Germplasm Resources in Cold and Arid Areas, Crop Cultivation and Genetic Improvement Key Laboratory of Inner Mongolia, Inner Mongolia Agricultural University, Hohhot, China; 2 Vocational and Technical College, Inner Mongolia Agricultural University, Baotou, China

**Keywords:** hybrid, ion content, maize, salt stress, yield

## Abstract

Salt-alkaline soils are one of the major abiotic factors limiting global crop production, and maize is a moderately salt-sensitive crop. With global warming and the widespread use of drip irrigation, maize cultivation faces severe challenges from saline-alkali stress in arid areas. We used nine spring maize hybrids as experimental materials to measure 24 agronomic and physiological traits at the seedling, flowering, and maturity stages in soil with a total salt content of 0.18%–0.61% and a pH of 9.0–9.2 by depth. Salt tolerance and yield evaluations of the tested maize hybrids were performed using principal component analysis, the membership function method, and GGE biplot analysis. The results identified root-to-shoot ratio, leaf Na^+^/K^+^ ratio, shoot dry weight and root Na^+^/K^+^ ratio as key traits for selecting hybrids with excellent productivity and stability under saline conditions. Additionally, GGE biplot analysis indicated Dika159 (H7) demonstrated superior productivity and stability in all test environments. These results facilitate the development of salt-tolerant maize germplasm and the breeding of stable and adaptable hybrids for saline-alkaline soils.

## Highlights

- We conducted a large-scale investigation across three growth stages and evaluated 24 traits to assess the salt tolerance of maize hybrids in soil with a total salt content of 0.18%–0.61% and a pH of 9.0–9.2 by depth.
- We identified root-to-shoot ratio,,leaf Na⁺/K⁺ ratio, shoot dry weight, and root Na⁺/K⁺ ratio as 4 key traits for selecting maize hybrids with excellent productivity and stability under saline conditions.

## 1 Introduction

Salt-alkali soil is extensively distributed, covering a vast area and encompassing various types. According to data from the Food and Agriculture Organization (FAO), soil salinization affects over 42.4 million hectares of surface soil (0–30 cm) and 83.3 million hectares of subsurface soil (30–100 cm) (Kumar et al., 2020). However, maize is a moderately salt-sensitive crop (Muhammad et al., 2015), and in salt-alkali land, maize yields are typically below 4.5 t/ha, just 70% of the average yield (Satir and Berberoglu, 2016). The improvement of salt-alkali soils and developing salt-tolerant crop varieties are critical and priorities in global agricultural research. To enhance global maize production and ensure food security, it is essential to breed salt-tolerant hybrids and utilize available saline-alkali land effectively.

Maize is one of the three major cereal crops in the world and serves as an important raw material for animal feed, food processing, and various industrial applications. Salt stress significantly affects maize growth and development at the seedling stage, primarily through the inhibition of plant growth and by altering metabolic and physiological processes within the plant (Gao et al., 2024). Under low salt stress, chlorophyll content and SPAD values increase, enhancing photosynthesis and promoting dry matter accumulation (Huqe et al., 2021). As the salt concentration of soil continues to rise, the impact of salt stress on plants become more pronounced under moderate and high salt stress, with chlorophyll content and SPAD values decreasing, leading to weakened photosynthesis and a significant reduction in dry matter accumulation compared to the no-salt treatment (Nabi et al., 2025; Hu et al., 2022; Tester and Davenport, 2003) Overall, the response exhibits a trend of initial increase followed by a decrease. Plant shoots are more sensitive to Na^+^ than roots, resulting in a greater reduction in shoot growth compared with root growth and consequently an increased root-to-shoot ratio. Salt stress inhibits maize growth in the field by reducing plant height, stem diameter, leaf area index, and other agronomic traits, and also decreases organic matter accumulation, ultimately leading to a significant reduction in yield, particularly under high salinity conditions (Ibrahim et al., 2023). Current research on maize primarily focuses on the germination and seedling stages, with relatively few studies addressing the maturity stage; therefore, further research is needed to investigate the effects of salt stress on yield. Moreover, breeding salt-tolerant maize varieties is essential for improving the utilization of saline–alkali soils.

Several methods are currently employed to assess salt tolerance in crops. Previous studies have applied membership function analysis to evaluate crop performance under abiotic stress, such as drought tolerance (Zhao et al., 2022; Min et al., 2016), water-logging tolerance (Wang et al., 2023), and salt tolerance (Tian et al., 2024). Additional analytical methods include Principal Component Analysis (PCA) (Ding et al., 2023), cluster analysis (Li et al., 2019), and Genotype Plus Genotype-By-Environment Interaction Biplot (GGE) (Li et al., 2023), among others. The combined approach of membership functions and PCA for comprehensive evaluation has been widely applied in crop studies (Miao et al., 2022), such as rice (Yano et al., 2019), wheat (Tabbita et al., 2023), sorghum (Rajabi Dehnavi et al., 2024), and sunflower (Ma et al., 2016). This study aims to assess the salt tolerance of nine spring maize hybrids under salt stress and to identify key traits for selecting salt tolerance maize hybrids during the seedling, flowering, and maturity stages, providing a comprehensive evaluation of maize’s salt tolerance. The ultimate goal is to propose effective selection traits and evaluation methods for breeding salt-tolerant spring maize hybrids.

## 2 Materials and methods

### 2.1 Experimental materials

This experiment used self-bred hybrid combinations (H1-H6) and commercial hybrids (H7-H9) as experimental materials. Detailed information on these materials is provided in **Table 1**.

**Table 1:** The list of experimental hybrids. .

Number	Parental inbred lines	Number	Parental inbred lines	Number	Hybrids
H1	Gaofeng H12×Gaofeng 157	H4	Gaofeng451×Gaofeng2524	H7	DiKa 159
H2	Gaofeng H6×Gaofeng 157	H5	Gaofeng451×Gaofeng 157	H8	HeYu 187
H3	Gaofeng451×Gaofeng25	H6	Gaofeng451×Gaofeng2504	H9	JingKe 968

Notes: H1-H6: self-bred hybrid combinations; H7-H9: commercial hybrids.

### 2.2 Experimental sites

**Figure 1:**
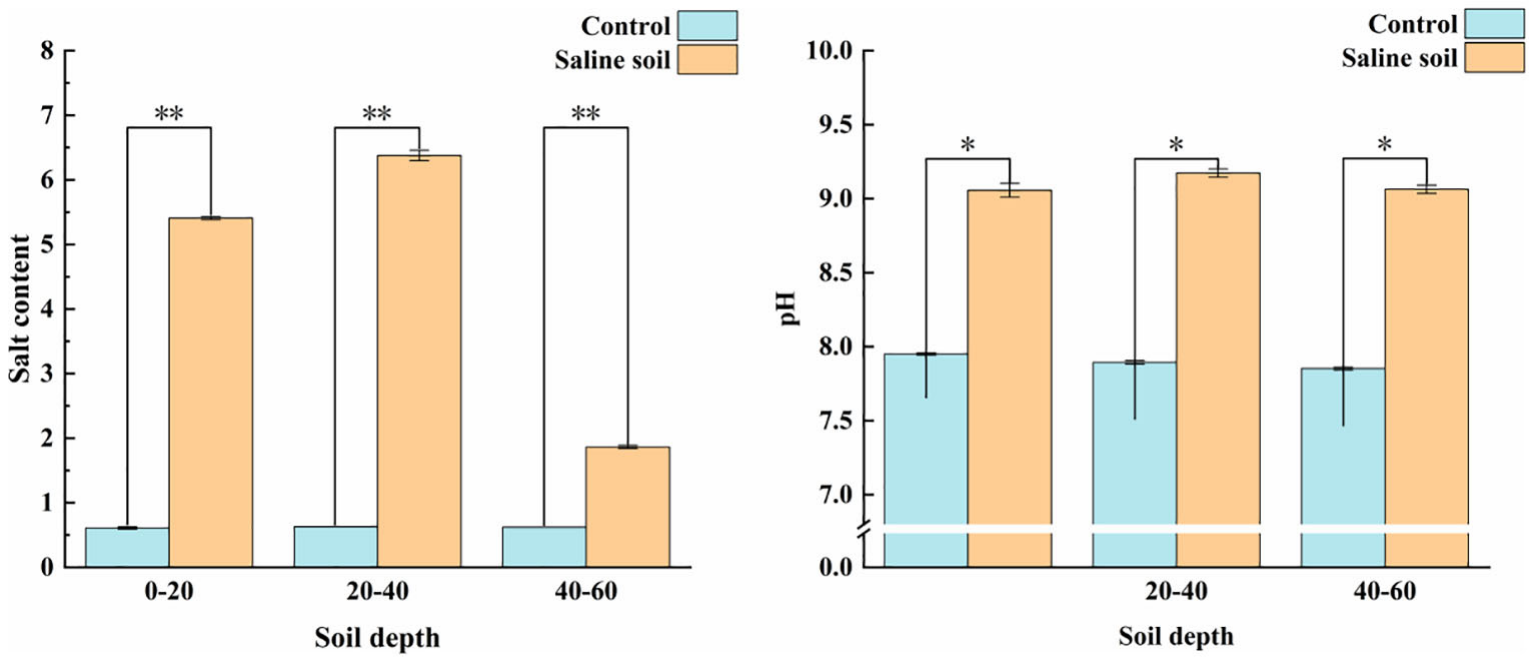
Soil salinity and pH (0–60 cm) at the experimental sites. Significant difference: *P<0.05; **P<0.01.

The control experimental site was located in Salaqi, Tumote Right County, Inner Mongolia, while the salt-alkali treatment experimental site was located in Wayao, Tumote Right County, Inner Mongolia. The Salaqi site is characterized by fertile soil and has hosted annual high-yield maize cultivation competitions for many years. In contrast, the Wayao site represents a typical saline-alkaline soil area in western Inner Mongolia. The two sites are approximately 19 km apart and share a continental semi-arid monsoon climate, with an average annual sunshine of 3095 hours, and an average annual precipitation of 346 mm (**Supplementary Figure 1**). The previous crop at both sites was maize. According to the soil agrochemical analysis, the Salaqi is classified as non-salt soil (Lu, 1999), with total salt content 0.04%-0.06% and pH 7.8-7.9 by soil depth, organic matter 26.17-26.44 g/kg, while Wayao is considered moderately salt-alkali soil, with total salt content 0.18%-0.61%, pH 9.0-9.2 by soil depth, organic matter 5.46-5.64 g/kg (**Figure 1**; **Supplementary Table 1**).

### 2.3 NaCl stress on maize during the three-leaf seedling stage

The experimental soil was collected from the top 10–20 cm of the cultivated layer in Salaqi. The soil was air-dried at room temperature and passed through a 1 mm sieve prior to use. The experiment was conducted in plastic pots. Maize seeds were soaked in distilled water for 24 hours, then treated with a 10% hydrogen peroxide solution for 10 minutes, followed by rinsing with distilled water 3 times. Five seeds were sown per pot, and after sowing, water was added to each pot until the soil reached the field water-holding capacity to ensure normal seedling emergence. When seedlings were at the three-leaf stage, 150 mmol/L NaCl solution was applied every 2 days for watering, with 200 ml of solution per pot. The control treatment was watered with distilled water. Salt stress was applied for 14 days, with a total salt content 0.32%, after which various traits were measured.

### 2.4 Field experiment design for hybrids under salt-alkali stress

A completely randomized block design with three repetitions was used for the experiment, with a planting density of 82,500 plants per hectare, a row length of 4.5 meters, a row spacing of 60 cm, and a subplot area of 27 m² comprising nine rows.

### 2.5 Salinity ion content in soil and maize plants

We measured Na^+^, K^+^, Ca^2+^, and Mg^2+^ concentrations both in the pre-sowing soil (0–30 cm layer) and in plant tissues. The target ions were extracted ultrasonically and analyzed by ion chromatography (Ma et al., 2019).

The 5-point sampling method is used to measure soil (0–60 cm layer) salinity. A soil extract was prepared at a 5:1 water-to-soil ratio, and its electrical conductivity (EC) value was measured with a conductivity meter. The total water-soluble salt content of the soil was calculated as follows (Abdelfattah et al., 2025):


Q=3.471L+0.015

Q = soil salinity, ‰

L = electrical conductivity of the soil sample at 25 °C, mS/cm

We used the same method to measure Na^+^ and K^+^ contents in maize first ear leaf and root samples at flowering time.

### 2.6 Root morphology traits measurement

Root samples were scanned using an Epson Perfection V700 Photo scanner. The images are saved with a resolution of 300 dpi. The root samples are placed in an acrylic tray with 2.0-3.0 mm of distilled water to minimize overlap. After scanning, the images were analyzed with Win RHIZO software to obtain the root length, root surface area, and root volume of maize.

### 2.7 Yield and associated traits measurement

From each treatment, the middle two rows were harvested after removing border plants. The total number of plants, barren plants, and double-ear plants within the harvested area were recorded. The weight of standard ears is calculated from the total weight of all harvested ears, and ear length and ear diameter are measured using a digital seed inspection system (LQ-MB2, National Engineering Research Center for Information Technology in Agriculture, China). Kernel moisture content was analyzed using a seed moisture tester (PM-8188-A, Kett Electric Laboratory, Japan) in triplicate and averaged. Yield was calculated with moisture content standardized to 14%. Biomass was measured by first blanching samples at 105 °C for 30 minutes, then drying them at 80 °C to constant weight before final weighing.


$$\text{Yield}(\text{t})=\text{density}\times (1+\text{double}-\text{ear plant}\%-\text{barren plant}\%)\times \text{kernel number per ear}\times 100-\text{kernel weight}(\text{g})\times 0.86/10^{8}$$

### 2.8 Membership function and principal component analysis based comprehensive trait assessment

#### 2.8.1 Salt tolerance coefficient and salt tolerance index


Salt Tolerance Coefficient=Stress Group/Control Group

Salt Tolerance Index=Salt Tolerance Coefficient×Average Yield under Salt Stress/Average Yield of all tested hybrids under salt stress

#### 2.8.2 PCA for salt tolerance evaluation

This paper uses the Z-score method for standardization, the formula for which is:


$$x=\frac{X-\mu }{\sigma }$$

X is the raw data, μ is the mean, and σ is the standard deviation.

PCA is used to assess the salt tolerance among maize hybrids, with salt tolerance coefficients of each trait serving as inputs for the analysis, and principal components with eigenvalues greater than 1 were extracted. The maximum absolute value of each principal component’s eigenvector was defined as the evaluation index for that component.

The scores for each principal component are calculated as follows:


$${\text{F}}_{\text{i}}={\text{w}}_{\text{i}1}{\text{X}}_{1}+{\text{w}}_{\text{i}2}{\text{X}}_{2}+\ldots +{\text{w}}_{\text{im}}\text{Xn}$$

$w_{im}=\frac{\theta_{m}}{\sqrt{\lambda_{i}}}$, represents the weight of each variable in the principal component, $\theta_{m}$ is the coefficient corresponding to each variable in the component matrix, and $\sqrt{\lambda_{i}}$ is the square root of the eigenvalue for the i principal component. X_n_ represents the standardized data values of each trait.

#### 2.8.3 Calculation of membership function values for traits

The membership function values for the composite traits among maize hybrids were calculated using the following formula (Jin et al., 2025):


$$\mu (Xi)=\frac{Xi-\text{X}\min}{\text{X}\max-\text{X}\min}$$

X_i_ is the score of the i composite trait, X_max_ and X_min_ are the maximum and minimum scores of each trait in each principal component. The weight of each composite indicator is calculated as:


$$Wj=\frac{Pj}{\sum_{j=1}^{n}Pj}\;\;\;\;j=1,2,\ldots ,n$$

W_j_ represents the weight of the j composite index, and P represents the contribution rate of the j composite index for each maize hybrid derived from the PCA.

#### 2.8.4 GGE biplot

The grain yield data collected from two experimental sites were organized into a three-column data table of genotype-environment-yield, where each value represents the average yield of the corresponding genotype in the respective environment, known as the phenotype value (Yger). The linear statistical model for GGE biplot analysis is presented as follows (Li et al., 2023):


$${\text{Y}}_{\text{ger}}=\text{μ}+{\text{β}}_{\text{e}}+\text{ρge}+\text{ϵger}+\sum \text{λnγgnδge}$$

Where Y_ger_ represents the yield value of genotype g in environment e for the rth replication; μ is the overall mean; β_e_ represents the main effect of environment e; ρge is the residual of genotype g in environment e; ϵger represents the overall error; λn is the singular value of the nth principal component; γgn is the genotype g’s score for the nth eigenvector; δge is the environment e’s score for the nth eigenvector. The parameters λnγgn and γnδen are defined as the GGE principal component scores for genotype g and environment e, respectively, also known as IPCAn or PCn. The data analysis was performed using Microsoft Excel 365 and Genstat 23 software on the Windows operating system.

### 2.9 Data analysis

All data were processed using Microsoft Excel 2021 (Microsoft, United States) and are presented as mean ± standard error. We conducted a completely randomized block analysis of variance followed by Tukey’s honestly significant difference (HSD) test. Membership function were calculated using SPSS 27.0 (SPSS Institute Inc., United States).

## 3 Results

### 3.1 Effects of salt stress on traits of maize hybrids

Significant differences were observed among nine hybrids of all 24 measured traits over the two-year study. However, biomass and SPAD value showed no significant differences between treatments (**Table 2**; **Supplementary Tables 2**, **3**). Under salt stress, all hybrids exhibited significant reductions in nearly all measured morphological and physiological traits, with the exception of the root-to-shoot ratio. Concurrently, the hybrids increased Na^+^ content while decreasing K^+^ content in both shoots and roots under salt stress compared to control plants (**Supplementary Table 4**). The coefficient of variation for the traits under salt stress ranged from 1.97% to 56.89% (**Table 2**), indicating considerable variation in sensitivity to salt stress across different traits.

**Table 2:** Descriptive statistics and variance analysis for traits under salt stress.

Trait	Control		Salt stress		F value		
	Mean	CV	Mean	CV	Variety	Stress	Variety×stress
Seedling PH(cm)	50.65	8.91%	37.51	7.83%	216.07**	6.85**	1.50
SDW (g)	0.5405	14.38%	0.3946	19.98%	83.82**	9.66**	1.09
RDW (g)	0.2258	18.79%	0.1906	16.86%	13.95**	5.19**	1.91
RSR	0.42	15.09%	0.49	15.12%	16.76**	4.68**	2.19*
RL (cm)	1329.79	30.31%	979.29	18.30%	54.01**	8.17**	10.48**
RS (cm^2^)	181.35	23.94%	143.00	13.74%	33.8**	5.96**	5.53**
RV (cm^3^)	1.99	19.42%	1.72	18.69%	11.27**	5.79**	2.82**
Shoot Na^+^ (mg/g)	8.52	26.43%	18.15	15.36%	16007.77**	260.61**	232.02**
Shoot Na^+^/K^+^	2.71	32.61%	13.97	56.89%	532.86**	25.87**	33.9**
Root Na^+^ (mg/g)	18.69	12.50%	21.16	4.93%	1348.65**	243.17**	79.29**
Root Na^+^/K^+^	4.19	12.94%	4.36	5.06%	84.56**	181.42**	35.99**
PH (cm)	293.35	5.39%	201.64	3.01%	3049.84**	16.83**	7.78**
EH (cm)	142.78	10.77%	84.33	2.90%	1901.14**	13.92**	16.7**
Biomass (g)	270.87	9.54%	130.04	11.25%	62.2**	0.25	0.33
SPAD value	59.43	2.83%	49.67	2.49%	318.94**	1.49	1.93
LAI	5.71	10.40%	4.12	9.96%	238.84**	8.75**	1.97*
Leaf Na^+^ (mg/g)	4.24	7.42%	5.15	7.28%	4737.07**	199.73**	107.09**
Leaf Na^+^/K^+^	0.94	8.64%	1.25	8.61%	2221.01**	52.71**	41.26**
Yield (t/hm^2^)	13.63	12.20%	5.78	14.23%	893.36**	5.62**	3.03**
No. of ears for harvest	35.09	10.15%	24.56	14.62%	116.55**	4.3**	1.84
Ear weight (kg)	8.89	8.08%	3.68	12.12%	703.4**	2.33*	0.93
100-kernel weight (g)	42.35	6.96%	34.85	4.96%	121.43**	2.52*	1.83
Ear length (cm)	20.13	3.17%	18.12	3.19%	144.91**	4.68**	1.94
Ear diameter (cm)	5.61	3.10%	5.06	1.97%	1588.41**	14.7**	5.21**
Kernel moisture (%)	20.95	6.34%	16.91	4.20%	2.35	8.97**	2.76

*, ** indicated significance at P<0.01 and P<0.05, respectively. The same below. CV, Coefficient of Variation; Seedling PH, Seedling Plant Height; SDW, Shoot Dry Weight; RDW, Root Dry Weight; RSR, Root-to-Shoot Ratio.

### 3.2 Clustering of maize hybrids and agronomic traits under salt stress

**Figure 2:**
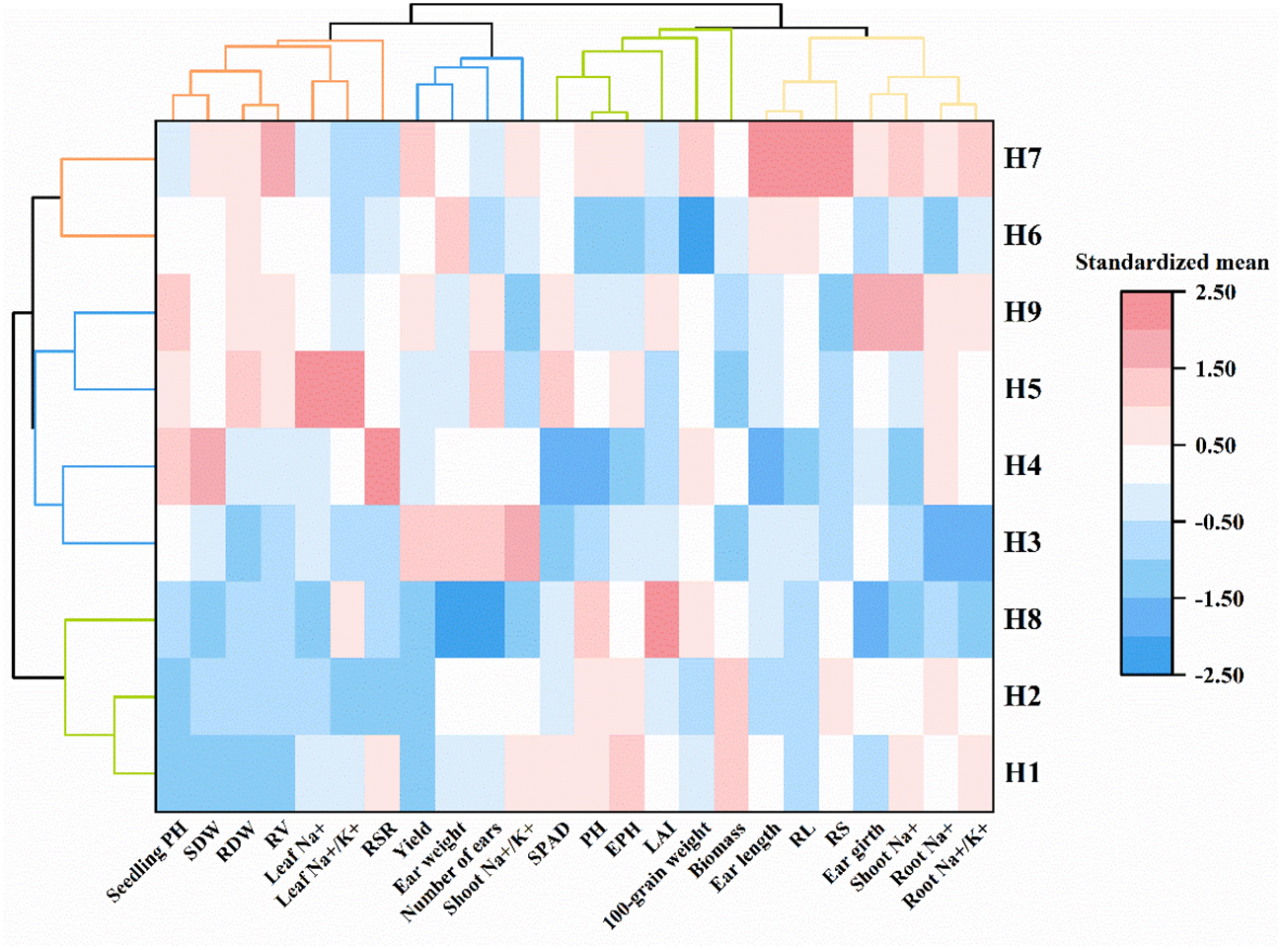
The hierarchical clustering heat map of traits and hybrids.

Following standardization of hybrid mean values for all traits, clustering analysis was conducted to assess the salt tolerance of the hybrids and identify trait–response associations. The nine hybrids were partitioned into three groups based on phenotypic differences: H6 and H7 formed a salt-tolerant group; H3, H4, H5, and H9 were moderately tolerant; and H1, H2, and H8 were classified as salt-sensitive (**Figure 2**). The 24 traits were categorized into four groups, containing 7, 4, 6, and 7 traits, respectively. Highly correlated traits, including seedling height, shoot dry weight, root dry weight, and root volume were assigned to the first group. Yield and ear weight clustered in the second category, plant height and ear height in the third category, and root length, root surface area, Na^+^ content in root, and root Na^+^/K^+^ ration in the fourth category (**Figure 2**). The first category primarily represented agronomic traits at the seedling stage, the second focused on yield and its related components, the third included traits at the flowering stage, and the fourth comprised root morphological traits and ion content.

The hierarchical clustering and heat map illustrates the relationships between nine maize hybrids and 24 different traits under salt stress. Each column represents a trait, while each row represents a maize hybrid. Darker red indicates higher values under salt stress, whereas darker blue indicates lower values under salt stress. The hybrids are classified into three groups, and the traits are categorized into four groups.

SDW: Shoot Dry Weight, RDW: Root Dry Weight, RSR: Root-Shoot Ratio, RL: Root Length, RS: Root Surface area, RV: Root Volume, PH: Plant Height, EH: Ear Height, LAI: Leaf Area Index.

### 3.3 Performance of key salt-tolerant traits in hybrids

**Figure 3:**
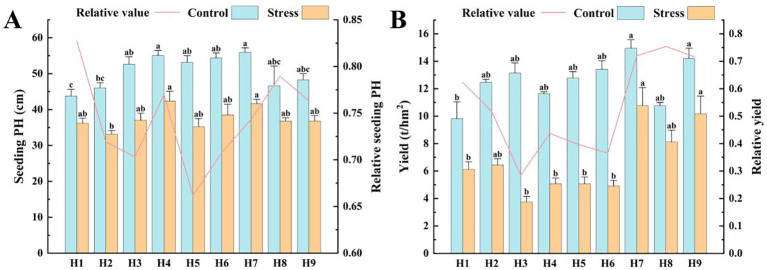
Multiple comparisons and relative values of seedling plant height and yield. **(A)** Seeding plant height(PH); **(B)** Yield. Different lowercase letters within the same color indicate significant differences at the p < 0.05 level.

We analyzed the seeding plant height and kernel yield as indicators of early vigor and yield potential, respectively. Under both control and salt conditions, seedling plant height of hybrids H7 and H4 differed significantly from that of H2. H5 exhibited the lowest relative value, indicating it was the most sensitive hybrid in terms of seedling plant height. (**Figure 3A**). Under stress conditions, with significant differences compared to H1, H3, H4, H5, and H6 (**Figure 3B**). H8 showed the highest relative yield, followed by H7, indicating these hybrids were least affected by saline stress. In contrast, H3 exhibited a 68% yield reduction, representing the greatest decrease under saline conditions (**Figure 3B**).

### 3.4 Principal component analysis, membership function analysis, and comprehensive evaluation of salt tolerance indicators

PCA was performed on the salt tolerance coefficients of 24 traits (**Supplementary Tables 5**, **6**). Six principal components (PC) with eigenvalues exceeding than 1 were extracted, accounting for a cumulative contribution rate of 93.62%. The first, second and third principal component explained 34.30%, 16.84% and 15.67% of the variance, respectively, with the root-to-shoot ratio, SPAD value, leaf Na^+^/K^+^ ratio showing the highest loading value. The fourth, fifth and sixth principal component accounted for 11.35%, 9.54% and 5.92% of the variance, respectively, with shoot dry weight, root Na^+^/K^+^ ratio and biomass exhibiting the highest loading value (**Supplementary Table 6**). Since biomass and SPAD values showed no significant differences between treatments (**Table 2**), the four key traits for selecting hybrids with excellent productivity and stability under saline conditions were root-to-shoot ratio, leaf Na^+^/K^+^ ratio, shoot dry weight and root Na^+^/K^+^ ratio. Among them, shoot dry weight is positive indicators, while root-to-shoot ratio, leaf Na^+^/K^+^ ratio and root Na^+^/K^+^ ratio are negative indicators. Linear regression of four key salt-tolerance traits produced a strong fit, with an R²score of 0.972.(**Supplementary Figure 5**). The hybrids H6, H8, and H7 ranked highest in comprehensive score, indicating their strong tolerance to salt stress (**Supplementary Table 7**).

### 3.5 G×E interaction GGE biplot analysis

**Figure 4:**
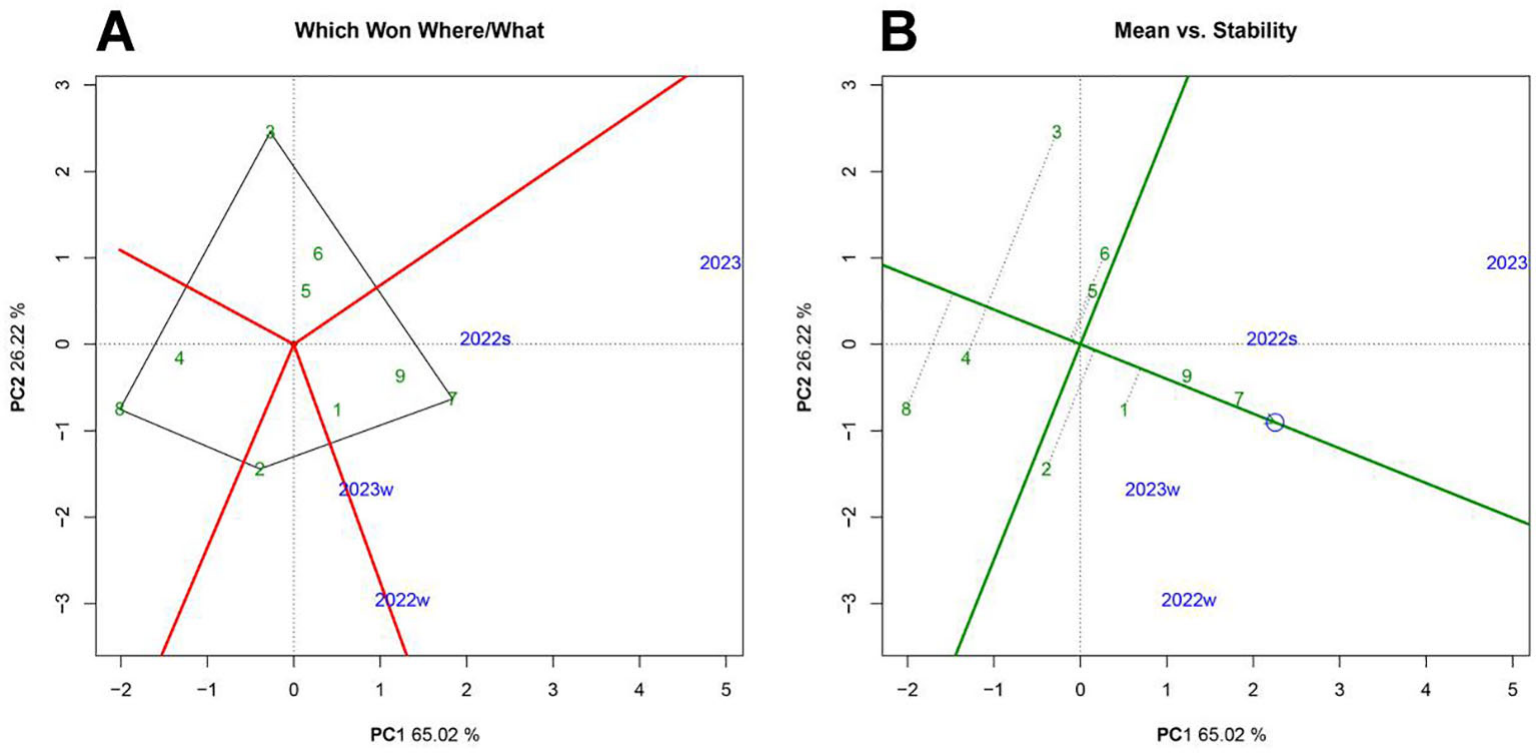
GGE biplot of two-year yield data for the tested hybrids. **(A)** “Which-won-where” view. Polygon vertices mark the top hybrids per sector. Dashed lines represent PC1 and PC2 zero axes. Hybrid codes: see **Table 1**. Environment codes: 2022s = Salaqi 2022, 2023s = Salaqi 2023, 2022w = Wayao 2022, 2023w = Wayao 2023. 2022s and 2023s were controls; 2022w and 2023w were saline-alkali lands **(B)** “Mean vs. stability” view. The arrowed line denotes the average environment axis. Genotype stability is inversely related to its perpendicular distance from this axis.

We utilized GGE Biplot to illustrate the relationship between genotype and environment. The biplot analysis accounts for 91.24% of the total observed variation, with the PC1 explaining 65.02% and PC2 explaining 26.22%. H2, H3, H7, and H8 are located at the vertices of the “which-win-where” polygon, indicating their superior performance in salt environments (**Figure 4A**). Among them, H7 exhibited the highest yield across all four growing seasons: 2022s, 2022w, 2023s, and 2023w. In productivity and stability analysis, H7 and H9 have projections closest to the Average Environment Coordinate (AEC) axis in the positive direction, indicating that H7 has a significantly higher yield than other hybrids (**Figure 4B**). Stability analysis of the hybrids reveals that H7 and H9 have the shortest perpendicular distance to the AEC axis, indicating the highest yield stability (**Figure 4B**; **Supplementary Table 8**). In contrast, H3 has the longest perpendicular distance to the AEC axis, suggesting the lowest yield stability.

## 4 Discussion

### 4.1 Salt stress impacts on agronomic and yield traits

Maize is moderately sensitive to salt stress, with cultivation in saline–alkali soils leading to significant yield reductions. Salt stress presents an increasing threat to crop productivity in arid regions. Consequently, the selection and breeding of salt-alkali tolerant maize hybrids have become key objectives in maize breeding programs aimed at improving yield stability in these environments. Identifying simple and effective selection criteria is a primary goal in salt-alkali tolerance breeding. Former studies have always focused on maize salt resistance at the seedling stage. In this study, we evaluated the salt tolerance of nine spring maize hybrids at the seedling and assessed their response to salt-alkali stress at flowering and maturity stages. Unfortunately, suitable experimental conditions for evaluating salt-alkali tolerance during the seedling stage were not available, but we continue to explore this area. Future research will focus on assessing salt-alkali tolerance throughout the entire growth period. We investigated 24 traits, which were the most comprehensive assessment for maize hybrids salt stress so far. Analysis of variance revealed highly significant differences among hybrids and between stress treatments for nearly all traits, indicating substantial genetic variation in salt tolerance among the tested maize hybrids (**Table 2**; **Figure 2**).Plant height, ear height, stem diameter, shoot dry weight, and root dry weight significantly reduced under salt stress in this study (**Table 2**; **Supplementary Tables 2**, **3**). Fresh weight and dry weight are key growth-related traits under salt stress, and the ability to maintain growth is widely recognized as a reliable indicator of salt tolerance, particularly during early vegetative stages (Negrão et al., 2017).

### 4.2 Roles of sodium and potassium ions under saline conditions

**Figure 5:**
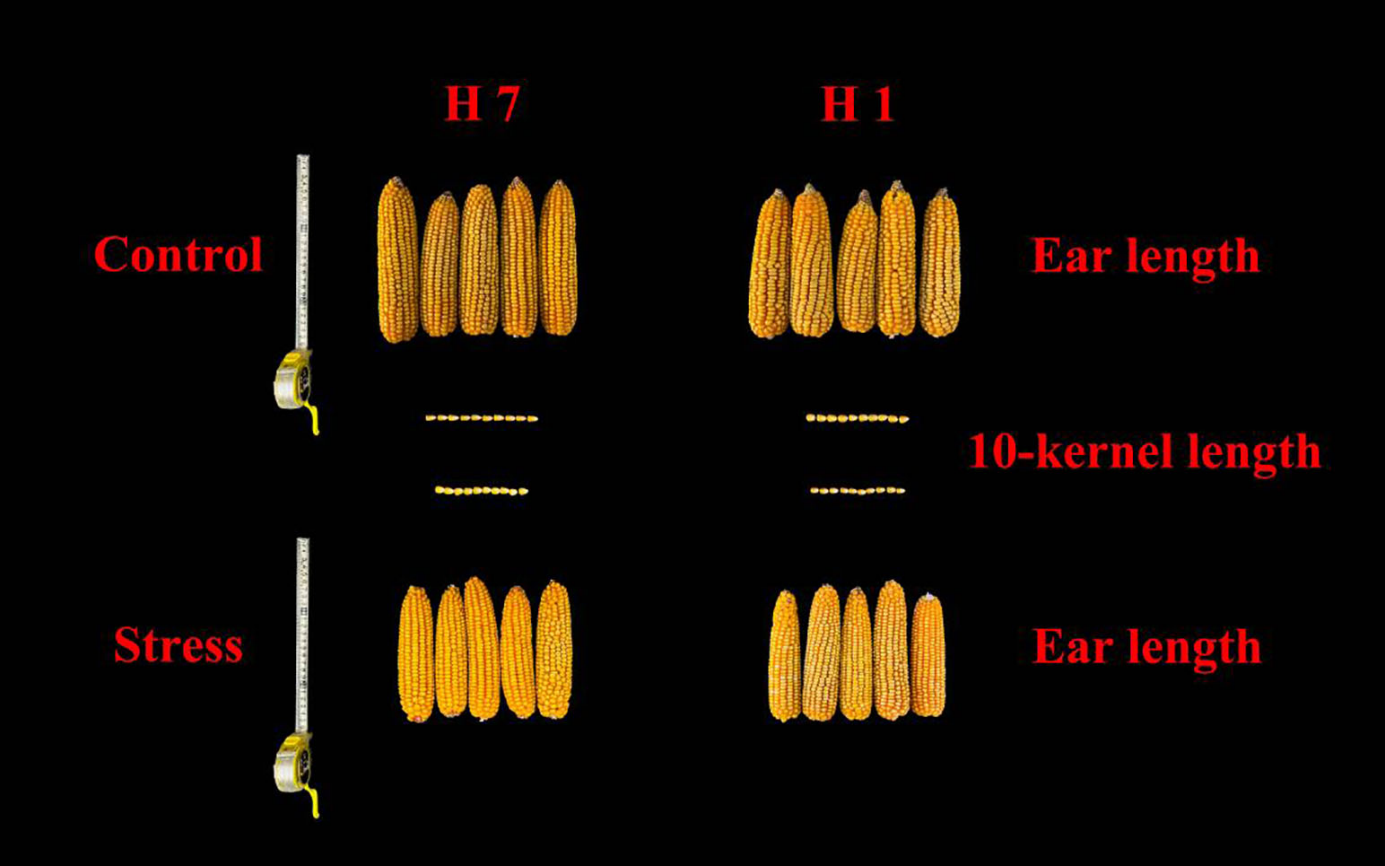
Salt alkali effect on the ear traits of hybrids H7 and H1.

When excessive salt ions accumulate in plant tissues, both vegetative and reproductive growth in maize are severely affected (Yang and Guo, 2018). This study found that the Na^+^/K^+^ ratio in both roots and leaves are crucial traits for evaluating salt tolerance in maize hybrids (**Figure 2**). Salt stress triggers excessive accumulation of Na^+^ in the roots, causing ion toxicity (Munns and Tester, 2008). Elevated Na^+^ disrupts K^+^ uptake and transport, impairing stomatal regulation and leading to water loss, necrosis, and reduced photosynthetic efficiency (Mahmood et al., 2024; Yang and Lu, 2005) (**Figure 5**; **Supplementary Figures 2**, **3**). Meanwhile, reproductive growth is also affected, leading to decreased pollen quality and fewer silks, ultimately resulting in lower yield (Gao et al., 2024). Currently cloned maize salt tolerance genes, such as *ZmHAK4* (Zhang et al., 2019), *ZmHKT1* (Zhang et al., 2018), *ZmHKT2* (Cao et al., 2019), *ZmHAK1* (Sheng et al, 2020), *ZmHAK11* (Liang et al., 2025), and *ZmSOS1* (Luo et al., 2025) are all involved in Na^+^/K^+^ and Na^+^/H^+^ transport. These genes promote shoot Na^+^ exclusion and enhance salt tolerance by retrieving Na^+^ from xylem sap (Zhang et al., 2019), highlighting that maintaining appropriate Na^+^ and K^+^ levels is a key traits for assessing salt tolerance. However, existing methods for measuring Na^+^ and K^+^ content are either complex or inefficient. Developing a simple and high-throughput method for quantifying these ions would greatly accelerate the breeding of salt-tolerant crop varieties. In the future, markers of salt-tolerant genes could be used to breed salt-tolerant maize hybrids (**Table 3**).

**Table 3:** Cloned salt-tolerance genes regulating sodium and potassium ion homeostasis in maize.

Gene	Gene ID	Chromosome	Annotation	Reference
*ZmHAK4*	*Zm00001d049987*	4	Root-to-shoot Na+ transportation	Zhang et al, 2019
*ZmHKT1*	*Zm00001d002378*	2	Regulating Na+ content in shoots	Zhang et al, 2018
*ZmHKT2*	*Zm00001d014680*	5	Regulating K+ content in shoots	Cao et al, 2019
*ZmHAK1*	*Zm00001d003861*	2	Regulating Na+ transport from shoots back to roots	Sheng et al, 2020
*ZmHAK11*	*Zm00001eb070910*	2	Regulating Na+ content in shoots	Liang et al., 2025
*ZmSOS1*	*Zm00001d031232*	1	Regulating Na+ transport to shoots	Luo et al., 2025

### 4.3 Key traits of maize under salt stress for breeding improvement

A key challenge in salt tolerance breeding is the rapid and accurate identification of salt-tolerant maize hybrids, which is essential for efficiently screening large hybrid populations. Most studies on maize salt tolerance have focused on a single growth stage, with limited attention to performance across multiple developmental stages. Previous studies used principal component scores combined with membership function weighting to calculate a comprehensive evaluation index (D-value) (Liu et al., 2024) identified salt tolerance index, single plant dry weight, SOD value, and root-to-shoot ratio as key traits for seedling-stage evaluation (Li et al., 2024). They proposed shoot fresh weight salt tolerance coefficient as the critical traits for evaluating maize inbred lines at the seedling stage. However, studies on salt tolerance evaluation at flowering and maturity stages are limited, and comprehensive assessments integrating multiple growth stages are rare. In this study, we evaluated salt tolerance across multiple growth stages and identified four key traits, including root-to-shoot ratio, leaf Na^+^/K^+^ ratio, shoot dry weight, and root Na^+^/K^+^ ratio. Correlation analysis displayed that yield was significantly or highly significantly positively correlated with root dry weight, root-to-shoot ratio, root volume, leaf Na^+^ content, and ear weight (**Supplementary Figure 4**). The root-to-shoot ratio followed this trend, with higher ratios associated with higher yield (**Figure 3A**).

### 4.4 Comprehensive evaluation of salt-tolerant maize hybrids

The top three hybrids ranked by salt tolerance index were H2, H7, and H8 (**Supplementary Table 7**). This discrepancy suggests that the evaluation systems for D-values and salt tolerance index are not fully aligned. The D-value considers all traits comprehensively, whereas the salt tolerance index focuses solely on yield under salt stress (**Supplementary Table 7**). By integrating both metrics, H7 and H8 were identified as stable salt-tolerant hybrids (**Supplementary Table 6**). H7 has strong lodging resistance, and its well-developed root system is likely a key factor in its notable tolerance to salt-alkali soils. However, the findings also indicated that hybrids with high comprehensive scores do not necessarily achieve the highest yield in salt-alkali soils. Thus, while comprehensive evaluation is valuable for identifying salt-tolerant maize hybrids, it alone is insufficient for selecting high-yielding hybrids under salt stress. Additional analytical methods are necessary to refine selection. Future work will incorporate molecular markers associated with salt resistance to screen hybrids. The lack of clear genotype–phenotype correlations in this study suggests that a larger set of markers or genome selection approaches may be necessary to effectively breed salt-tolerant hybrids. Nonetheless, this study provides a foundation for screening salt-tolerant maize hybrids and offers valuable insights for selecting germplasm suitable for saline–alkali land.

## 5 Conclusions

By evaluating nine spring maize hybrids across three growth stages, this study provides comprehensive insight into their salt tolerance performance. Treating these stages as an integrated system in principal component analysis allowed for the identification of four key traits for assessing salt tolerance: root-to-shoot ratio, leaf Na^+^/K^+^ ratio, shoot dry weight and root Na^+^/K^+^ ratio. Using a combined approach of principal component analysis and membership function analysis, the hybrids were ranked by salt tolerance as follows: H6 > H8 > H7 > H2 > H4 > H9 > H1 > H3 > H5. Furthermore, GGE biplot analysis integrated with yield performance revealed that H7 exhibited the highest productivity and greatest stability among the nine hybrids under salt stress.

## Data Availability

The original contributions presented in the study are included in the article/**Supplementary Material**. Further inquiries can be directed to the corresponding authors.
