# Supplementary material for: Comprehensive assessment of salt tolerance in nine spring maize hybrids across the entire growth period
Source: Front Plant Sci. 2026 Jul 20;17:1871695. doi: 10.3389/fpls.2026.1871695 (PMC13429399; doi:10.3389/fpls.2026.1871695)
Supplement: Supplementary file 1 [file SupplementaryFile1.docx]

Supplementary Table 1 Soil base productivity, soil salinity and ion content at the experimental sites

| Year | Test site | Available N  (mg/kg) | Available K  (mg/kg) | Available P  (mg/kg) | Organic matter  (g/kg) | Na^+^  (mg/kg) | K^+^  (mg/kg) | Ca^2+^  (mg/kg) | Mg^2+^  (mg/kg) |
| --- | --- | --- | --- | --- | --- | --- | --- | --- | --- |
| 2022 | Salaqi | 55.82 | 109.88 | 31.98 | 26.17 | 6.20 | 2.99 | 17.72 | 4.32 |
| 2022 | Wayao | 28.94 | 209.01 | 4.98 | 5.46 | 38.37 | 9.92 | 8.41 | 5.15 |
| 2023 | Salaqi | 56.91 | 109.58 | 32.71 | 26.44 | 4.52 | 2.55 | 20.31 | 4.76 |
| 2023 | Wayao | 27.05 | 176.35 | 5.11 | 5.64 | 45.22 | 7.92 | 8.82 | 9.36 |

Supplementary Table 2 Effect of salt stress on agronomic traits of maize at seedling stage

|  | Number | Plant height at seedling stage  (cm) | Shoot dry weight  (g) | Root dry weight  (g) | Root-shoot ratio | Root length  (cm) | Root surface  (cm^2^) | Root volume  (cm^3^) |
| --- | --- | --- | --- | --- | --- | --- | --- | --- |
| Control | H1 | 43.73±1.92c | 0.438±0.042b | 0.2056±0.0224b | 0.50±0.05a | 12.45±1.23bc | 160.38±16.2bc | 1.65±0.17c |
|  | H2 | 46.05±1.41bc | 0.5244±0.0354ab | 0.195±0.0164b | 0.38±0.03ab | 10.56±0.74c | 148.17±9.9c | 1.66±0.11c |
|  | H3 | 52.65±2.02ab | 0.5239±0.0409ab | 0.1832±0.0135b | 0.36±0.01ab | 12.02±0.94bc | 160.7±11.74c | 1.72±0.12bc |
|  | H4 | 55.05±1.39a | 0.6544±0.0321a | 0.2117±0.0164ab | 0.34±0.04b | 10.95±0.78c | 165.19±11.37bc | 1.99±0.14abc |
|  | H5 | 53.18±1.82ab | 0.5973±0.0334ab | 0.2977±0.0201a | 0.50±0.02a | 16.5±0.99b | 225.03±13.55ab | 2.44±0.15ab |
|  | H6 | 54.37±1.34ab | 0.5593±0.0221ab | 0.2697±0.0197ab | 0.48±0.03ab | 11.38±0.72c | 171.31±9.97bc | 2.06±0.12abc |
|  | H7 | 55.97±1.20a | 0.5811±0.0287ab | 0.2485±0.021ab | 0.44±0.04ab | 12.57±1.16bc | 176.16±15.3bc | 1.98±0.17abc |
|  | H8 | 46.61±5.47abc | 0.4075±0.0855b | 0.1721±0.0433b | 0.35±0.05ab | 10.38±2.47c | 146.06±36.07c | 1.64±0.42bc |
|  | H9 | 48.25±1.72abc | 0.5785±0.0452ab | 0.2491±0.0199ab | 0.44±0.03ab | 22.87±1.34a | 279.15±18.26a | 2.74±0.22a |
| Salt Stress | H1 | 36.17±1.41ab | 0.285±0.024d | 0.1436±0.0141b | 0.52±0.03ab | 11.2±0.82a | 138.67±11.32ab | 1.37±0.13b |
|  | H2 | 33.13±1.02b | 0.3036±0.016cd | 0.1624±0.0176ab | 0.52±0.05ab | 10.25±0.99a | 136.72±12.53ab | 1.5±0.16b |
|  | H3 | 37.01±1.97ab | 0.376±0.0318bcd | 0.1646±0.0158ab | 0.44±0.03ab | 9.76±0.9a | 142.69±15.5ab | 1.69±0.23ab |
|  | H4 | 42.38±2.69a | 0.5432±0.0258a | 0.1801±0.0155ab | 0.35±0.04b | 5.83±0.63b | 105.03±8.99b | 1.57±0.15ab |
|  | H5 | 35.19±2.29ab | 0.3971±0.0318bcd | 0.1798±0.024ab | 0.43±0.04ab | 8.88±1ab | 128.01±14.55ab | 1.48±0.18b |
|  | H6 | 38.49±3.02ab | 0.412±0.0203abcd | 0.2323±0.0143ab | 0.57±0.04a | 10.95±0.26a | 170.32±4.86a | 2.13±0.11ab |
|  | H7 | 41.59±1.28a | 0.4071±0.0269bc | 0.232±0.0175a | 0.58±0.03a | 10.08±0.78a | 165.89±9.41a | 2.26±0.17a |
|  | H8 | 36.80±0.90ab | 0.3604±0.0241bcd | 0.2026±0.0256ab | 0.54±0.05a | 12.05±1.14a | 149.89±15.06ab | 1.49±0.17b |
|  | H9 | 36.82±1.44ab | 0.4674±0.0372ab | 0.218±0.0197ab | 0.49±0.05ab | 9.13±0.77ab | 149.77±12.21ab | 1.96±0.16ab |

Notes: Different lowercase letters indicate significant differences at the *p* < 0.05 level, below.

Supplementary Table 3 Effect of salt stress on yield-related traits of maize

|  | Number | Plant height  (cm） | Ear height  (cm） | Biomass  （g） | SPAD value | Leaf area index | Number of ears harvested | Ear weight  (kg) | 100-kernel weight (g) | Ear length  （cm） | Ear diameter  （cm） |
| --- | --- | --- | --- | --- | --- | --- | --- | --- | --- | --- | --- |
| Control | 1 | 275.48±2.53d | 139.73±1.69b | 263.03±13.34b | 58.91±0.71bc | 6.06±0.14a | 36.67±2.28b | 9.26±0.41a | 47.39±1.24a | 20.24±0.19a | 6.22±0.03a |
|  | 2 | 281.05±2.91d | 145.97±1.36b | 268.98±14.79b | 57.03±0.51c | 5.76±0.17ab | 33.67±2.46c | 8.16±0.22b | 41.14±0.66ab | 19.02±0.22c | 5.78±0.04b |
|  | 3 | 320.78±3.16a | 164.7±1.19a | 267.46±16.93b | 58.90±0.62bc | 6.41±0.21a | 30.67±3.52a | 7.92±0.91b | 44.94±1.28ab | 20.61±0.24a | 5.56±0.04bc |
|  | 4 | 293.5±2.41c | 146.23±2.14b | 294.63±15.89a | 60.73±0.63ab | 6.09±0.20a | 35.51±1.48b | 8.83±0.58ab | 45.04±0.92bc | 20.11±0.14ab | 5.56±0.02cd |
|  | 5 | 302.83±2.19bc | 158.6±1.71a | 209.02±5.63c | 59.78±0.47abc | 6.13±0.12ab | 35.50±2.74b | 9.26±0.62a | 41.78±0.39bc | 19.6±0.19ab | 5.69±0.02cde |
|  | 6 | 292.63±2.56c | 138.2±2.06b | 292.5±18.97a | 60.93±0.52ab | 5.32±0.19bc | 40.67±2.30a | 9.61±0.47a | 37.59±1.15bc | 20.14±0.2ab | 5.42±0.03cde |
|  | 7 | 295.75±2.24c | 138.63±2.43b | 287.28±16.13a | 58.13±0.44c | 4.82±0.11c | 38.33±2.65a | 9.53±0.43a | 41.86±0.55bc | 20.87±0.18bc | 5.4±0.03def |
|  | 8 | 271.17±2.67d | 109.75±2.67c | 272.6±16.69b | 62.37±0.45a | 4.81±0.11c | 29.17±3.76d | 7.88±0.66b | 40.56±1.10c | 20.97±0.24bc | 5.57±0.04ef |
|  | 9 | 306.97±1.71b | 143.19±1.96b | 282.35±15.88ab | 58.07±0.43c | 6.04±0.19ab | 35.67±0.56a | 9.53±0.24a | 40.85±0.65c | 19.64±0.19c | 5.95±0.03f |
| Salt Stress | 1 | 198.24±4.65b | 82.84±3.24b | 132.56±10.61b | 50.11±2.09a | 4.46±0.34a | 26.33±1.45b | 3.81±0.56a | 36.65±0.98a | 17.64±0.51b | 5.01±0.06a |
|  | 2 | 192.84±3.38b | 81.72±3.31b | 108.11±7.52c | 49.73±1.38a | 4.22±0.34b | 31.67±2.40a | 4.41±0.44a | 32.8±2.05ab | 17.95±0.40b | 5.02±0.07ab |
|  | 3 | 203.16±5.29ab | 83.21±3.31b | 137.18±11.05b | 49.15±1.83ab | 4.79±0.37a | 19.05±1.73c | 2.99±0.14b | 33.55±0.75ab | 19.19±0.41a | 4.99±0.07ab |
|  | 4 | 199.16±5.88b | 82.12±4.10b | 121.13±12.68c | 48.72±1.86b | 4.18±0.33b | 24.67±1.45b | 3.22±0.45a | 33.46±1.87ab | 17.73±0.49b | 5.04±0.06ab |
|  | 5 | 200.82±6.55ab | 86.67±4.51a | 131.78±8.92b | 48.21±3.11b | 3.47±0.31c | 21.07±1.02c | 3.51±0.49a | 33.72±1.18ab | 18.06±0.39ab | 5.02±0.07ab |
|  | 6 | 198.92±3.93b | 87.92±2.55a | 116.55±5.33c | 52.32±2.26a | 3.99±0.27b | 26.02±2.13b | 3.62±0.27a | 37.67±1.16b | 17.55±0.56b | 5.02±0.08b |
|  | 7 | 211.28±3.19a | 81.84±2.72b | 121.94±10.15c | 50.28±1.34a | 3.74±0.21bc | 24.67±2.33b | 4.23±0.41a | 35.98±1.31b | 18.98±0.31a | 5.03±0.05b |
|  | 8 | 199.2±4.19b | 86.23±5.81a | 149.83±10.78a | 48.54±1.94b | 3.83±0.22bc | 22.67±4.18c | 3.6±0.73a | 33.85±0.4b | 18.06±0.45ab | 5.13±0.05b |
|  | 9 | 211.16±2.65a | 86.48±3.82a | 151.33±12.37a | 49.97±2.45ab | 4.43±0.33ab | 25.02±3.51b | 3.7±0.64a | 35.99±1.31b | 17.96±0.53b | 5.31±0.08b |

Supplementary Table 4 Na^+^ and K^+^ contents of maize at flowering time

|  | Number | Shoot Na^+^ content  (mg/g) | Shoot Na^+^/K^+^ ratio  (mg/g) | Root Na^+^ content  (mg/g) | Root Na^+^/K^+^ ratio | Leaf Na^+^ content  (mg/g) | Leaf Na^+^/K^+^ content  (mg/g) |
| --- | --- | --- | --- | --- | --- | --- | --- |
| Control | 1 | 11.41±0.09a | 3.52±0.05b | 19.94±0.01b | 4.14±0.03b | 3.86±0.05d | 0.88±0.01ef |
|  | 2 | 12.05±0.39a | 4.36±0.36a | 20.81±0.04a | 4.37±0.02a | 4.4±0.04b | 0.96±0.01bcd |
|  | 3 | 10.02±0.03b | 3.23±0.01b | 17.03±0.28c | 3.54±0.05c | 4.61±0.01a | 1.10±0.01a |
|  | 4 | 5.89±0.08e | 1.71±0.07de | 15.11±0.15d | 3.06±0.03d | 4.14±0.02c | 0.97±0.02bc |
|  | 5 | 7.71±0.01d | 2.36±0.07c | 15.59±0.15d | 3.17±0.03d | 3.77±0.04d | 0.85±0.01f |
|  | 6 | 6.07±0.04e | 1.63±0.02e | 20.39±0.11ab | 4.46±0.02a | 4.21±0.02c | 0.91±0.01def |
|  | 7 | 6.58±0.06e | 2.30±0.09cd | 17.81±0.07c | 3.57±0.02c | 4.7±0.02a | 1.01±0.01b |
|  | 8 | 8.21±0.05cd | 2.42±0.03c | 21.01±0.29a | 4.03±0.06b | 4.10±0.03c | 0.87±0.01ef |
|  | 9 | 8.74±0.03c | 2.91±0.05bc | 20.54±0.06ab | 4.42±0.02a | 4.38±0.01b | 0.92±0cde |
| Salt Stress | 1 | 20.05±0.09b | 12.13±0.08bcd | 22.62±0.22a | 5.69±0.07a | 5.17±0.03cd | 1.28±0.01b |
|  | 2 | 20.11±0.35b | 7.70±0.28cd | 21.03±0.21bcd | 4.42±0.04d | 5.14±0.01cd | 1.25±0.01bc |
|  | 3 | 12.45±0.24d | 10.34±0.83bcd | 20.70±0.16cde | 4.85±0.04b | 4.88±0.05e | 1.23±0.03bc |
|  | 4 | 21.52±0.04a | 14.16±0.22bc | 19.56±0.03f | 4.44±0.03d | 5.12±0.03d | 1.27±0.02bc |
|  | 5 | 17.21±0.19c | 15.88±0.73b | 20.37±0.06e | 4.41±0.02d | 5.21±0.01cd | 1.25±0.01bc |
|  | 6 | 16.45±0.13c | 33.03±1.20a | 20.62±0.06de | 4.54±0.01cd | 5.27±0.03bc | 1.44±0.02a |
|  | 7 | 16.44±0.21c | 16.06±0.26b | 21.44±0.04b | 4.83±0.03b | 5.76±0.01a | 1.30±0.01b |
|  | 8 | 19.88±0.03b | 5.85±0.13d | 22.83±0.07a | 4.86±0.04b | 4.38±0.02f | 1.03±0.01d |
|  | 9 | 19.24±0.12b | 10.54±0.03bcd | 21.22±0.03bc | 4.73±0.09bc | 5.41±0.02b | 1.19±0.01c |

Supplementary Table 5 Salt tolerance coefficient of investigated traits

| **Hybrid** | **Seedling PH** | **SDW** | **RDW** | **RSR** | **RL** | **RS** | **RV** | **Shoot Na^+^** | **Shoot Na^+^/K^+^** | **Root Na^+^** | **Root Na^+^/K^+^** | **PH** | **EH** | **Biomass** | **SPAD value** | **LAI** | **Leaf Na^+^** | **Leaf Na^+^/K^+^** | **Yield** | **Number of ears harvested** | **Ear weight** | **100-kernel weight** | **Ear length** | **Ear diameter** |
| --- | --- | --- | --- | --- | --- | --- | --- | --- | --- | --- | --- | --- | --- | --- | --- | --- | --- | --- | --- | --- | --- | --- | --- | --- |
| **H1** | **0.83** | **0.65** | **0.70** | **1.05** | **0.90** | **0.86** | **0.83** | **1.76** | **3.45** | **1.13** | **0.83** | **0.72** | **0.59** | **0.50** | **0.85** | **0.74** | **1.34** | **1.46** | **0.45** | **0.68** | **0.40** | **0.80** | **0.81** | **0.89** |
| **H2** | **0.72** | **0.58** | **0.83** | **1.38** | **0.97** | **0.92** | **0.90** | **1.67** | **1.77** | **1.01** | **1.00** | **0.69** | **0.56** | **0.40** | **0.87** | **0.73** | **1.17** | **1.30** | **0.52** | **0.79** | **0.47** | **0.80** | **0.85** | **0.84** |
| **H3** | **0.70** | **0.72** | **0.90** | **1.23** | **0.81** | **0.89** | **0.98** | **1.24** | **3.20** | **1.22** | **0.89** | **0.63** | **0.51** | **0.51** | **0.83** | **0.75** | **1.06** | **1.11** | **0.28** | **0.55** | **0.30** | **0.73** | **0.76** | **0.81** |
| **H4** | **0.77** | **0.83** | **0.85** | **1.02** | **0.53** | **0.64** | **0.78** | **3.65** | **8.34** | **1.30** | **0.89** | **0.68** | **0.56** | **0.41** | **0.80** | **0.69** | **1.23** | **1.31** | **0.44** | **0.79** | **0.36** | **0.78** | **0.78** | **0.84** |
| **H5** | **0.66** | **0.66** | **0.6** | **0.86** | **0.54** | **0.57** | **0.60** | **2.23** | **6.73** | **1.31** | **0.94** | **0.66** | **0.55** | **0.63** | **0.81** | **0.57** | **1.38** | **1.48** | **0.40** | **0.61** | **0.36** | **0.82** | **0.79** | **0.80** |
| **H6** | **0.71** | **0.74** | **0.86** | **1.18** | **0.96** | **0.99** | **1.03** | **2.71** | **20.25** | **1.01** | **0.99** | **0.68** | **0.64** | **0.40** | **0.86** | **0.75** | **1.26** | **1.59** | **0.37** | **0.67** | **0.36** | **0.95** | **0.75** | **0.82** |
| **H7** | **0.74** | **0.70** | **0.93** | **1.32** | **0.80** | **0.94** | **1.14** | **2.50** | **6.98** | **1.20** | **0.89** | **0.71** | **0.59** | **0.42** | **0.87** | **0.78** | **1.23** | **1.29** | **0.44** | **0.63** | **0.43** | **0.85** | **0.84** | **0.85** |
| **H8** | **0.79** | **0.88** | **1.18** | **1.53** | **1.16** | **1.03** | **0.91** | **2.42** | **2.42** | **1.09** | **0.90** | **0.73** | **0.79** | **0.55** | **0.78** | **0.79** | **1.07** | **1.19** | **0.51** | **0.87** | **0.44** | **0.82** | **0.96** | **0.86** |
| **H9** | **0.76** | **0.81** | **0.88** | **1.11** | **0.40** | **0.54** | **0.72** | **2.20** | **3.63** | **1.03** | **0.96** | **0.69** | **0.60** | **0.54** | **0.86** | **0.73** | **1.23** | **1.29** | **0.43** | **0.81** | **0.42** | **0.85** | **0.84** | **0.82** |

Supplementary Table 6 Principal component contribution rate and feature vector of investigated traits.

| Traits | PCA1 | PCA2 | PCA3 | PCA4 | PCA5 | PCA6 |
| --- | --- | --- | --- | --- | --- | --- |
| Seedling PH | 0.616 | -0.335 | 0.237 | -0.202 | 0.500 | -0.190 |
| SDW | 0.336 | -0.419 | -0.022 | 0.783 | 0.133 | -0.119 |
| RDW | 0.819 | -0.054 | -0.328 | 0.443 | -0.071 | -0.069 |
| RSR | 0.881 | 0.199 | -0.341 | 0.032 | -0.218 | 0.021 |
| RL | 0.699 | 0.351 | -0.196 | -0.109 | 0.111 | 0.471 |
| RS | 0.671 | 0.523 | -0.306 | -0.047 | 0.201 | 0.313 |
| RV | 0.494 | 0.667 | -0.386 | 0.043 | 0.284 | -0.087 |
| Shoot Na^+^ | 0.018 | -0.133 | 0.511 | 0.641 | 0.315 | -0.327 |
| Shoot Na^+^/K^+^ | -0.247 | 0.650 | 0.335 | 0.570 | 0.263 | 0.083 |
| Root Na^+^ | -0.559 | -0.483 | -0.236 | 0.091 | 0.401 | 0.016 |
| Root Na^+^/K^+^ | -0.083 | 0.471 | 0.251 | 0.239 | -0.789 | -0.077 |
| PH (cm) | 0.767 | -0.141 | 0.528 | -0.182 | 0.218 | 0.074 |
| EH (cm) | 0.772 | -0.136 | 0.254 | 0.390 | -0.013 | 0.400 |
| Biomass | -0.282 | -0.625 | -0.050 | -0.008 | -0.242 | 0.590 |
| SPAD value | -0.044 | 0.711 | 0.142 | -0.483 | -0.130 | -0.330 |
| LAI | 0.811 | 0.321 | -0.297 | 0.017 | 0.203 | -0.185 |
| Leaf Na^+^ | -0.561 | 0.028 | 0.730 | -0.250 | 0.229 | 0.140 |
| Leaf Na^+^/K^+^ | -0.359 | 0.413 | 0.750 | 0.004 | 0.194 | 0.293 |
| Yield | 0.633 | -0.269 | 0.557 | -0.262 | -0.196 | -0.089 |
| Number of ears harvested | 0.640 | -0.375 | 0.429 | 0.191 | -0.293 | -0.269 |
| Ear weight | 0.681 | -0.039 | 0.458 | -0.362 | -0.353 | -0.119 |
| 100-kernel weight | 0.116 | 0.593 | 0.625 | 0.371 | -0.056 | 0.172 |
| Ear length | 0.808 | -0.421 | 0.095 | -0.058 | -0.285 | 0.179 |
| Ear diameter | 0.650 | -0.148 | 0.230 | -0.443 | 0.544 | -0.011 |
| Eigenvalue | 8.233 | 4.042 | 3.760 | 2.723 | 2.289 | 1.422 |
| Contribution rate (%) | 34.304 | 16.840 | 15.667 | 11.347 | 9.538 | 5.924 |
| Cumulative contribution rate (%) | 34.304 | 51.145 | 66.811 | 78.158 | 87.696 | 93.620 |
| Weight | 0.366 | 0.180 | 0.167 | 0.121 | 0.102 | 0.063 |

Notes:Seedling PH (Seedling Plant Height), SDW (Shoot Dry Weight), RDW (Root Dry Weight), RSR (Root-Shoot Ratio), RL(Root Length), RS(Root Surface), RV(Root Volume), PH(Plant Height), EH (Ear Height), LAI(Leaf Area Index).

Supplementary Table 7 Evaluation parameters for tested hybrids: F value, membership function value, and ranking (based on D value or salt tolerance index)

| Number | F1 | F2 | F3 | F4 | F5 | F6 | U1 | U2 | U3 | U4 | U5 | U6 | D value | Salt tolerance index | D value ranking | Index ranking |
| --- | --- | --- | --- | --- | --- | --- | --- | --- | --- | --- | --- | --- | --- | --- | --- | --- |
| H1 | 1.67 | 1.69 | 2.48 | 1.86 | 1.61 | 0.68 | 0.63 | 0.07 | 0.19 | 0.08 | 0.17 | 0.35 | 0.32 | 0.50 | 6 | 4 |
| H2 | 2.08 | 1.30 | 2.01 | 1.29 | 1.00 | 0.48 | 0.83 | 0.00 | 0.06 | 0.00 | 0.00 | 0.21 | 0.33 | 0.61 | 5 | 1 |
| H3 | 1.70 | 1.67 | 1.78 | 1.78 | 1.36 | 0.66 | 0.65 | 0.06 | 0.00 | 0.07 | 0.10 | 0.34 | 0.29 | 0.19 | 7 | 9 |
| H4 | 1.13 | 2.90 | 3.76 | 4.52 | 2.74 | 0.17 | 0.37 | 0.27 | 0.52 | 0.46 | 0.48 | 0.00 | 0.38 | 0.41 | 4 | 6 |
| H5 | 0.88 | 2.46 | 3.30 | 3.26 | 2.09 | 0.72 | 0.25 | 0.19 | 0.40 | 0.28 | 0.30 | 0.38 | 0.28 | 0.37 | 9 | 7 |
| H6 | 0.36 | 7.38 | 5.56 | 8.24 | 4.63 | 1.62 | 0.00 | 1.00 | 1.00 | 1.00 | 1.00 | 1.00 | 0.63 | 0.33 | 1 | 8 |
| H7 | 1.59 | 2.93 | 3.02 | 3.51 | 2.31 | 0.59 | 0.59 | 0.27 | 0.33 | 0.32 | 0.36 | 0.29 | 0.41 | 0.53 | 3 | 2 |
| H8 | 2.44 | 1.27 | 2.14 | 2.12 | 1.32 | 0.51 | 1.00 | 0.00 | 0.10 | 0.12 | 0.09 | 0.23 | 0.42 | 0.51 | 2 | 3 |
| H9 | 1.59 | 1.49 | 2.66 | 2.34 | 1.40 | 0.22 | 0.59 | 0.04 | 0.23 | 0.15 | 0.11 | 0.04 | 0.29 | 0.48 | 7 | 5 |

Supplementary Table 8 Yield performance of tested hybrids across two sites and two years.

| Hybrids | Salaqi (t/hm^2^) | | Wayao (t/hm^2^) | |
| --- | --- | --- | --- | --- |
|  | 2022 | 2023 | 2022 | 2023 |
| H1 | 12.90 | 14.17 | 6.09 | 6.13 |
| H2 | 12.30 | 12.66 | 6.04 | 6.86 |
| H3 | 12.27 | 14.01 | 2.86 | 4.62 |
| H4 | 11.84 | 11.63 | 5.12 | 5.05 |
| H5 | 13.40 | 13.73 | 4.54 | 5.61 |
| H6 | 12.31 | 14.54 | 4.28 | 5.53 |
| H7 | 13.74 | 16.15 | 6.02 | 7.13 |
| H8 | 10.87 | 10.65 | 5.07 | 5.86 |
| H9 | 12.79 | 15.60 | 6.58 | 5.22 |
| mean | 12.49 | 13.68 | 5.18 | 5.78 |
| max | 14.89 | 16.65 | 9.08 | 8.35 |
| min | 9.94 | 10.50 | 2.49 | 3.42 |
| sd | 1.35 | 1.74 | 1.51 | 1.43 |
| CV(%) | 0.11 | 0.13 | 0.29 | 0.25 |

Supplementary Table 9 Yield components of tested hybrids across two sites in 2023

|  | Number | Ear number | Ear weight（kg） | Kernel weight（kg) | Kernel moisture | 100-kernel weight (g) |
| --- | --- | --- | --- | --- | --- | --- |
| Control | H1 | 36.67 | 9.26 | 7.08 | 0.22 | 47.39 |
|  | H2 | 33.67 | 8.16 | 6.54 | 0.23 | 41.14 |
|  | H3 | 30.67 | 7.92 | 6.57 | 0.22 | 44.94 |
|  | H4 | 35.50 | 8.83 | 6.46 | 0.21 | 45.04 |
|  | H5 | 35.50 | 9.26 | 7.39 | 0.20 | 41.78 |
|  | H6 | 40.67 | 9.61 | 7.76 | 0.20 | 37.59 |
|  | H7 | 38.33 | 9.53 | 8.16 | 0.19 | 41.86 |
|  | H8 | 29.17 | 7.88 | 5.91 | 0.20 | 40.56 |
|  | H9 | 35.67 | 9.53 | 8.17 | 0.21 | 40.85 |
| Salt Stress | H1 | 26.33 | 3.81 | 3.02 | 0.16 | 36.65 |
|  | H2 | 30.00 | 4.41 | 3.44 | 0.18 | 32.80 |
|  | H3 | 19.00 | 2.99 | 2.29 | 0.17 | 33.55 |
|  | H4 | 24.67 | 3.22 | 2.53 | 0.18 | 33.46 |
|  | H5 | 21.00 | 3.51 | 2.79 | 0.17 | 33.72 |
|  | H6 | 26.00 | 3.62 | 2.70 | 0.16 | 37.67 |
|  | H7 | 24.67 | 4.23 | 3.53 | 0.17 | 35.98 |
|  | H8 | 22.67 | 3.60 | 2.91 | 0.17 | 33.85 |
|  | H9 | 25.00 | 3.70 | 2.63 | 0.18 | 35.99 |


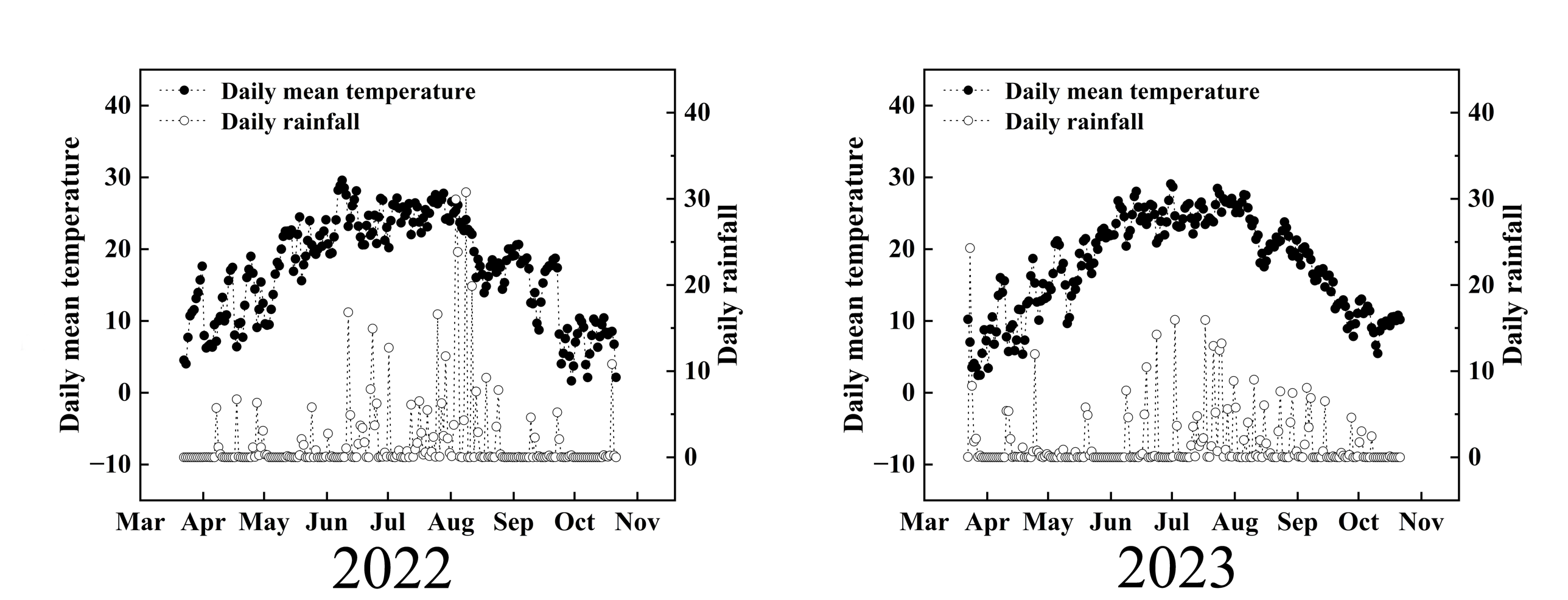


Supplementary Figure 1 Main meteorological factors at the test sites across two years.





Supplementary Figure 2 Morphological characteristics of tested hybrids under control conditions at seedling stage.





Supplementary Figure 3 Morphological characteristics of tested hybrids under salt stress at seedling stage.

Supplementary Figure 4 Correlation analysis of investigated traits


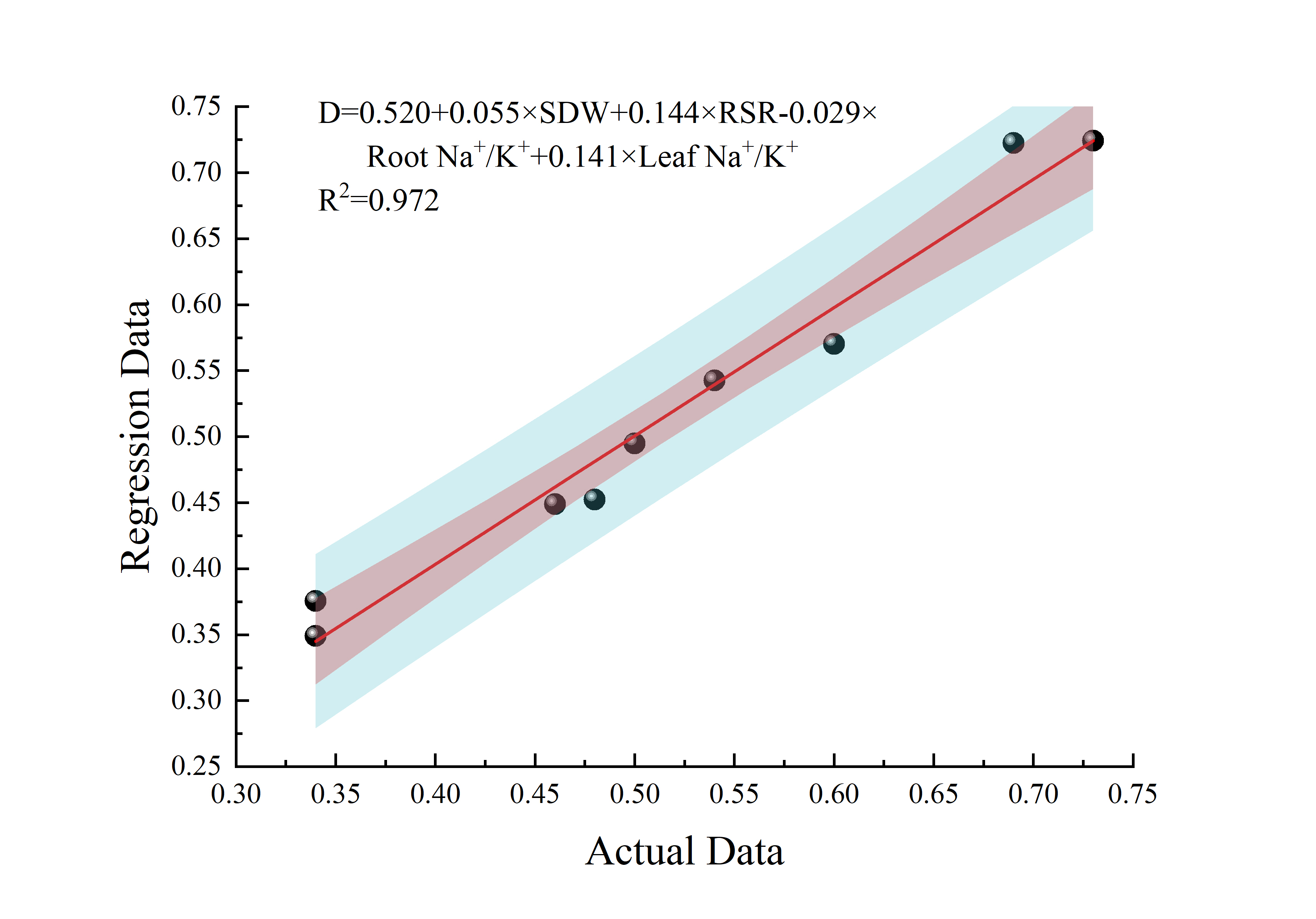


******

Supplementary Figure 5 Linear regression of four key traits for salt tolerance





Supplementary Figure 6 Ear and kernel traits of tested hybrids under control conditions.





Supplementary Figure 7 Ear and kernel traits of tested hybrids under salt stress.
